# Supplementary material for: Methylsulfonylmethane Improves Knee Quality of Life in Participants with Mild Knee Pain: A Randomized, Double-Blind, Placebo-Controlled Trial
Source: Nutrients. 2023 Jun 30;15(13):2995. doi: 10.3390/nu15132995 (PMC10346176; doi:10.3390/nu15132995)
Supplement: Supplementary file 1 [file nutrients-15-02995-s001.zip › nutrients-2455333-supplementary.pdf]

Supplementary Table S1. Comparison of the amount of change in JKOM and JOA scores at each week from week 0.

			0 to 4 week				0 to 8 week				0 to 12 week			
			MSM group	Placebo group	difference	*P* value[1]	MSM group	Placebo group	difference	*P* value[1]	MSM group	Placebo group	difference	*P* value[1]
		(Unit)	Mean ± SD	Mean ± SD	(95% CI)		Mean ± SD	Mean ± SD	(95% CI)		Mean ± SD	Mean ± SD	(95% CI)	
JKOM	Ⅰ:VAS	(amount of change)	-3.8 ± 26.0	-8.4 ± 28.7	1.8 (-7.5 to 11.2)	0.696	-12.6 ± 26.3	-12.3 ± 28.1	-3.2 (-12.6 to 6.1)	0.493	-13.5 ± 26.8	-16.1 ± 25.5	0.2 (-9.1 to 9.5)	0.966
	Ⅱ:Pain and stiffiness in knees	(amount of change)	-1.6 ± 3.1	-1.5 ± 3.9	-0.3 (-1.6 to 1.0)	0.659	-2.7 ± 3.1	-2.3 ± 3.7	-0.6 (-1.8 to 0.7)	0.388	-4.1 ± 2.9	-3.3 ± 3.3	-0.1 (-2.3 to 0.3)	0.123
	Ⅲ:Condition in daily life	(amount of change)	-1.4 ± 3.7	-1.3 ± 3.7	-0.2 (-1.5 to 1.1)	0.745	-1.9 ± 2.7	-1.9 ± 3.5	-0.2 (-1.4 to 1.1)	0.803	-2.8 ± 3.0	-2.6 ± 2.7	-0.4 (-1.7 to 0.8)	0.493
	Ⅳ:General activities	(amount of change)	-0.9 ± 1.6	-1.5 ± 2.1	0.1 (-0.6 to 0.7)	0.806	-1.4 ± 2.2	-1.8 ± 1.9	-0.1 (-0.8 to 0.5)	0.66	-1.9 ± 2.4	-2.3 ± 2.4	-0.3 (-1.0 to 0.3)	0.280
	Ⅴ:Health conditions	(amount of change)	-0.7 ± 1.0	-0.7 ± 1.3	-0.1 (-0.6 to 0.3)	0.556	-0.9 ± 1.0	-0.9 ± 1.0	-0.1 (-0.5 to 0.4)	0.693	-1.3 ± 1.1	-0.9 ± 1.3	-0.5 (-0.9 to 0.0)	0.032*
	Total JKOM score	(amount of change)	-4.6 ± 6.1	-4.9 ± 8.9	-0.2 (-3.1 to 2.7)	0.891	-6.9 ± 6.1	-6.9 ± 7.9	-0.8 (-3.7 to 2.1)	0.588	-10.2 ± 7.0	-9.0 ± 7.0	-2.1 (-5.0 to 0.7)	0.144
JOA	Total score	(amount of change)	-0.5 ± 6.5	0.6 ± 5.4	-1.1 (-3.2 to 1.0)	0.303	0.6 ± 6.7	0.7 ± 4.7	-0.1 (-2.1 to 2.0)	0.954	0.5 ± 7.4	0.8 ± 6.2	-0.2 (-2.3 to 1.8)	0.817

CI, confidence interval; [1]Comparison between groups using a linear mixed model with baseline values as covariates, time points, groups, time and group interactions, baseline values and time point interactions, and study participants as factors. * $p < 0.05$ between the MSM and placebo groups.

Supplementary Table S2. Comparison of the amount of change in IL-1β, IL-6, Hs-CRP and CIIC at each week from week 0.

		0 to 4 week				0 to 8 week				0 to 12 week			
		MSM group	Placebo group	difference	*P* value[1]	MSM group	Placebo group	difference	*P* value[1]	MSM group	Placebo group	difference	*P* value[1]
	(Unit)	Mean ± SD	Mean ± SD	(95% CI)		Mean ± SD	Mean ± SD	(95% CI)		Mean ± SD	Mean ± SD	(95% CI)	
IL-1β	(ammount of change)	0.0 ± 0.1	0.0 ± 0.0	0.0 (0.0 to 0.0)	0.790	0.0 ± 0.1	0.0 ± 0.0	0.0 (0.0 to 0.0)	0.889	0.0 ± 0.2	0.0 ± 0.1	0.0 (0.0 to 0.0)	0.191
IL-6		0.4 ± 0.7	0.4 ± 0.8	0.0 (-0.2 to 0.3)	0.758	0.2 ± 0.5	0.4 ± 0.5	-0.2 (-0.5 to 0.1)	0.155	0.2 ± 0.7	0.3 ± 0.5	-0.1 (-0.3 to 0.2)	0.651
Hs-CRP		0.0 ± 0.2	0.1 ± 0.5	0.0 (-0.2 to 0.1)	0.502	0.0 ± 0.1	0.0 ± 0.2	0.0 (-0.2 to 0.1)	0.590	0.0 ± 0.4	0.0 ± 0.2	0.1 (-0.1 to 0.2)	0.400
CⅡC		9.8 ± 37.6	21.0 ± 42.7	-1.9 (-12.3 to 8.5)	0.722	-25.8 ± 28.9	-21.5 ± 25.6	2.8 (-7.6 to 13.2)	0.597	-9.9 ± 28.9	-4.9 ± 33.1	1.7 (-8.7 to 12.1)	0.743

CI, confidence interval; [1]Comparison between groups using a linear mixed model with baseline values as covariates, time points, groups, time and group interactions, baseline values and time point interactions, and study participants as factors.

Supplementary Table S3. Urine Analysis of Safety evaluation

Inspection item		period	MSM group			Placebo group			group comparison *				
			n	number of eligible paticipants	Percentage of eligible paticipants (%)	n	number of eligible paticipants	Percentage of eligible paticipants (%)	⊿ (%)	95% CI-	95% CI+	χ^2	P
urine	protein	4 week	44	0	0.0	44	1	2.3	-2.3	-6.7	2.2	1.011	1.000
		8 week	43	1	2.3	44	1	2.3	0.1	-6.2	6.4	0.000	1.000
		12 week	43	1	2.3	44	2	4.5	-2.2	-9.9	5.4	0.322	1.000
	glucose	4 week	44	0	0.0	44	0	0.0	0.0	N.A.	N.A.	N.A.	N.A.
		8 week	43	0	0.0	44	1	2.3	-2.3	-6.8	2.2	0.989	1.000
		12 week	43	0	0.0	44	1	2.3	-2.3	-6.8	2.2	0.989	1.000
	urobilinogen	4 week	44	1	2.3	44	0	0.0	2.3	-2.2	6.7	1.011	1.000
		8 week	43	0	0.0	44	0	0.0	0.0	N.A.	N.A.	N.A.	N.A.
		12 week	43	0	0.0	44	0	0.0	0.0	N.A.	N.A.	N.A.	N.A.
	bilirubin	4 week	44	0	0.0	44	0	0.0	0.0	N.A.	N.A.	N.A.	N.A.
		8 week	43	0	0.0	44	0	0.0	0.0	N.A.	N.A.	N.A.	N.A.
		12 week	43	0	0.0	44	0	0.0	0.0	N.A.	N.A.	N.A.	N.A.
	pH	4 week	44	1	2.3	44	0	0.0	2.3	-2.2	6.7	1.011	1.000
		8 week	43	1	2.3	44	0	0.0	2.3	-2.2	6.8	1.035	0.494
		12 week	43	0	0.0	44	1	2.3	-2.3	-6.8	2.2	0.989	1.000
	occult blood	4 week	44	3	6.8	44	5	11.4	-4.5	-16.6	7.5	0.550	0.713
		8 week	43	4	9.3	44	2	4.5	4.8	-5.9	15.4	0.766	0.434
		12 week	43	7	16.3	44	2	4.5	11.7	-1.1	24.5	3.228	0.089

n: number ;⊿: between-group difference (test food group - placebo group);95% CI -: 95% lower confidence interval/95% CI +: 95% upper confidence interval/chi 2: chi-square value; P: significant probability; 4 week: at test after 4 weeks of intake; 8 week: at test after 8 weeks of intake; 12 week : at test after 12 weeks of intake; N.A.: Not Available; *: comparison between groups using chi-square test

Supplementary Table S4. Blood analysis of safety evaluation (1)

Inspection item		period	MSM group			Placebo group			group comparison *				
			n	number of eligible paticipants	Percentage of eligible paticipants (%)	n	number of eligible paticipants	Percentage of eligible paticipants (%)	⊿ (%)	95% CI-	95% CI+	χ^2	P
blood	ketone bodies	4 week	44	1	2.3	44	0	0.0	2.3	-2.2	6.7	1.011	1.000
		8 week	43	1	2.3	44	2	4.5	-2.2	-9.9	5.4	0.322	1.000
		12 week	43	0	0.0	44	0	0.0	0.0	N.A.	N.A.	N.A.	N.A.
	wlite blood cells	4 week	44	0	0.0	44	1	2.3	-2.3	-6.7	2.2	1.011	1.000
		8 week	43	1	2.3	44	3	6.8	-4.5	-13.3	4.3	1.001	0.616
		12 week	43	1	2.3	44	3	6.8	-4.5	-13.3	4.3	1.001	0.616
	red blood cells	4 week	44	1	2.3	44	1	2.3	0.0	-6.2	6.2	0.000	1.000
		8 week	43	4	9.3	44	1	2.3	7.0	-2.8	16.8	1.984	0.202
		12 week	43	2	4.7	44	1	2.3	2.4	-5.3	10.0	0.370	0.616
	hemoglobin	4 week	44	2	4.5	44	0	0.0	4.5	-1.7	10.8	2.047	0.494
		8 week	43	2	4.7	44	0	0.0	4.7	-1.6	10.9	2.095	0.241
		12 week	43	1	2.3	44	2	4.5	-2.2	-9.9	5.4	0.322	1.000
	hematocrit	4 week	44	2	4.5	44	3	6.8	-2.3	-11.9	7.4	0.212	1.000
		8 week	43	2	4.7	44	2	4.5	0.1	-8.7	8.9	0.001	1.000
		12 week	43	5	11.6	44	3	6.8	4.8	-7.3	17.0	0.603	0.484
	plaelet count	4 week	44	2	4.5	44	3	6.8	-2.3	-11.9	7.4	0.212	1.000
		8 week	43	1	2.3	44	4	9.1	-6.8	-16.5	3.0	1.838	0.360
		12 week	43	0	0.0	44	2	4.5	-4.5	-10.8	1.8	2.001	0.494
	MCV	4 week	44	0	0.0	44	1	2.3	-2.3	-6.7	2.2	1.011	1.000
		8 week	43	1	2.3	44	1	2.3	0.1	-6.2	6.4	0.000	1.000
		12 week	43	0	0.0	44	1	2.3	-2.3	-6.8	2.2	0.989	1.000
	MCH	4 week	44	0	0.0	44	0	0.0	0.0	N.A.	N.A.	N.A.	N.A.
		8 week	43	2	4.7	44	0	0.0	4.7	-1.6	10.9	2.095	0.241
		12 week	43	0	0.0	44	1	2.3	-2.3	-6.8	2.2	0.989	1.000
	MCHC	4 week	44	0	0.0	44	1	2.3	-2.3	-6.7	2.2	1.011	1.000
		8 week	43	0	0.0	44	0	0.0	0.0	N.A.	N.A.	N.A.	N.A.
		12 week	43	1	2.3	44	1	2.3	0.1	-6.2	6.4	0.000	1.000
	neutrophil rate	4 week	44	0	0.0	44	1	2.3	-2.3	-6.7	2.2	1.011	1.000
		8 week	43	0	0.0	44	0	0.0	0.0	N.A.	N.A.	N.A.	N.A.
		12 week	43	1	2.3	44	0	0.0	2.3	-2.2	6.8	1.035	0.494
	lymphocyte rate	4 week	44	0	0.0	44	1	2.3	-2.3	-6.7	2.2	1.011	1.000
		8 week	43	0	0.0	44	1	2.3	-2.3	-6.8	2.2	0.989	1.000
		12 week	43	1	2.3	44	0	0.0	2.3	-2.2	6.8	1.035	0.494
	monocyte rate	4 week	44	0	0.0	44	1	2.3	-2.3	-6.7	2.2	1.011	1.000
		8 week	43	0	0.0	44	0	0.0	0.0	N.A.	N.A.	N.A.	N.A.
		12 week	43	1	2.3	44	0	0.0	2.3	-2.2	6.8	1.035	0.494
	eosinophil rate	4 week	44	1	2.3	44	0	0.0	2.3	-2.2	6.7	1.011	1.000
		8 week	43	1	2.3	44	1	2.3	0.1	-6.2	6.4	0.000	1.000
		12 week	43	0	0.0	44	0	0.0	0.0	N.A.	N.A.	N.A.	N.A.
	basophil rate	4 week	44	0	0.0	44	0	0.0	0.0	N.A.	N.A.	N.A.	N.A.
		8 week	43	0	0.0	44	0	0.0	0.0	N.A.	N.A.	N.A.	N.A.
		12 week	43	0	0.0	44	0	0.0	0.0	N.A.	N.A.	N.A.	N.A.
	AST (GOT)	4 week	44	0	0.0	44	0	0.0	0.0	N.A.	N.A.	N.A.	N.A.
		8 week	43	0	0.0	44	0	0.0	0.0	N.A.	N.A.	N.A.	N.A.
		12 week	43	0	0.0	44	0	0.0	0.0	N.A.	N.A.	N.A.	N.A.
	ALT (GPT)	4 week	44	0	0.0	44	0	0.0	0.0	N.A.	N.A.	N.A.	N.A.
		8 week	43	0	0.0	44	1	2.3	-2.3	-6.8	2.2	0.989	1.000
		12 week	43	0	0.0	44	0	0.0	0.0	N.A.	N.A.	N.A.	N.A.
	γ-GT (γ-GTP)	4 week	44	2	4.5	44	1	2.3	2.3	-5.3	9.9	0.345	1.000
		8 week	43	2	4.7	44	1	2.3	2.4	-5.3	10.0	0.370	0.616
		12 week	43	1	2.3	44	0	0.0	2.3	-2.2	6.8	1.035	0.494
	ALP	4 week	44	0	0.0	44	1	2.3	-2.3	-6.7	2.2	1.011	1.000
		8 week	43	1	2.3	44	2	4.5	-2.2	-9.9	5.4	0.322	1.000
		12 week	43	1	2.3	44	1	2.3	0.1	-6.2	6.4	0.000	1.000
	LD (LDH)	4 week	44	4	9.1	44	9	20.5	-11.4	-26.2	3.5	2.256	0.229
		8 week	43	5	11.6	44	4	9.1	2.5	-10.3	15.3	0.151	0.739
		12 week	43	5	11.6	44	6	13.6	-2.0	-16.0	12.0	0.079	1.000
	LAP	4 week	44	3	6.8	44	2	4.5	2.3	-7.4	11.9	0.212	1.000
		8 week	43	3	7.0	44	2	4.5	2.4	-7.4	12.2	0.237	0.676
		12 week	43	1	2.3	44	1	2.3	0.1	-6.2	6.4	0.000	1.000
	tota bilirubin	4 week	44	2	4.5	44	1	2.3	2.3	-5.3	9.9	0.345	1.000
		8 week	43	3	7.0	44	0	0.0	7.0	-0.7	14.6	3.179	0.116
		12 week	43	3	7.0	44	0	0.0	7.0	-0.7	14.6	3.179	0.116
	direct bilirubin	4 week	44	0	0.0	44	0	0.0	0.0	N.A.	N.A.	N.A.	N.A.
		8 week	43	0	0.0	44	0	0.0	0.0	N.A.	N.A.	N.A.	N.A.
		12 week	43	0	0.0	44	0	0.0	0.0	N.A.	N.A.	N.A.	N.A.

n: number ;⊿: between-group difference (test food group - placebo group);95% CI -: 95% lower confidence interval/95% CI +: 95% upper confidence interval/chi 2: chi-square value; P: significant probability; 4 week: at test after 4 weeks of intake; 8 week: at test after 8 weeks of intake; 12 week : at test after 12 weeks of intake; N.A.: Not Available; *: comparison between groups using chi-square test

Supplementary Table S5. Blood analysis of safety evaluation (2)

Inspection item		period	MSM group			Placebo group			group comparison *				
			n	number of eligible paticipants	Percentage of eligible paticipants (%)	n	number of eligible paticipants	Percentage of eligible paticipants (%)	⊿ (%)	95% CI-	95% CI+	χ^2	P
	indirect bilirubin	4 week	44	2	4.5	44	2	4.5	0.0	-8.7	8.7	0.000	1.000
		8 week	43	2	4.7	44	1	2.3	2.4	-5.3	10.0	0.370	0.616
		12 week	43	3	7.0	44	0	0.0	7.0	-0.7	14.6	3.179	0.116
	cholinesterase (ChE)	4 week	44	0	0.0	44	1	2.3	-2.3	-6.7	2.2	1.011	1.000
		8 week	43	0	0.0	44	1	2.3	-2.3	-6.8	2.2	0.989	1.000
		12 week	43	1	2.3	44	0	0.0	2.3	-2.2	6.8	1.035	0.494
	total protein	4 week	44	1	2.3	44	0	0.0	2.3	-2.2	6.7	1.011	1.000
		8 week	43	2	4.7	44	0	0.0	4.7	-1.6	10.9	2.095	0.241
		12 week	43	1	2.3	44	1	2.3	0.1	-6.2	6.4	0.000	1.000
	urea nitrogen	4 week	44	2	4.5	44	4	9.1	-4.5	-15.1	6.0	0.715	0.676
		8 week	43	1	2.3	44	6	13.6	-11.3	-22.7	0.1	3.760	0.110
		12 week	43	1	2.3	44	3	6.8	-4.5	-13.3	4.3	1.001	0.616
	creatinine	4 week	44	0	0.0	44	4	9.1	-9.1	-17.8	-0.4	4.190	0.116
		8 week	43	1	2.3	44	3	6.8	-4.5	-13.3	4.3	1.001	0.616
		12 week	43	0	0.0	44	1	2.3	-2.3	-6.8	2.2	0.989	1.000
	uric acid	4 week	44	1	2.3	44	1	2.3	0.0	-6.2	6.2	0.000	1.000
		8 week	43	0	0.0	44	1	2.3	-2.3	-6.8	2.2	0.989	1.000
		12 week	43	1	2.3	44	1	2.3	0.1	-6.2	6.4	0.000	1.000
	CK	4 week	44	5	11.4	44	1	2.3	9.1	-1.4	19.6	2.862	0.202
		8 week	43	3	7.0	44	2	4.5	2.4	-7.4	12.2	0.237	0.676
		12 week	43	2	4.7	44	5	11.4	-6.7	-18.1	4.7	1.324	0.434
	sodium	4 week	44	2	4.5	44	1	2.3	2.3	-5.3	9.9	0.345	1.000
		8 week	43	1	2.3	44	2	4.5	-2.2	-9.9	5.4	0.322	1.000
		12 week	43	0	0.0	44	0	0.0	0.0	N.A.	N.A.	N.A.	N.A.
	patassium	4 week	44	3	6.8	44	2	4.5	2.3	-7.4	11.9	0.212	1.000
		8 week	43	0	0.0	44	3	6.8	-6.8	-14.5	0.9	3.037	0.241
		12 week	43	2	4.7	44	2	4.5	0.1	-8.7	8.9	0.001	1.000
	crawl	4 week	44	1	2.3	44	1	2.3	0.0	-6.2	6.2	0.000	1.000
		8 week	43	2	4.7	44	1	2.3	2.4	-5.3	10.0	0.370	0.616
		12 week	43	0	0.0	44	1	2.3	-2.3	-6.8	2.2	0.989	1.000
	calcium	4 week	44	0	0.0	44	0	0.0	0.0	N.A.	N.A.	N.A.	N.A.
blood		8 week	43	0	0.0	44	0	0.0	0.0	N.A.	N.A.	N.A.	N.A.
		12 week	43	0	0.0	44	0	0.0	0.0	N.A.	N.A.	N.A.	N.A.
	inorganic phosphorus	4 week	44	2	4.5	44	3	6.8	-2.3	-11.9	7.4	0.212	1.000
		8 week	43	2	4.7	44	2	4.5	0.1	-8.7	8.9	0.001	1.000
		12 week	43	2	4.7	44	1	2.3	2.4	-5.3	10.0	0.370	0.616
	serum iron	4 week	44	2	4.5	44	1	2.3	2.3	-5.3	9.9	0.345	1.000
		8 week	43	1	2.3	44	2	4.5	-2.2	-9.9	5.4	0.322	1.000
		12 week	43	4	9.3	44	1	2.3	7.0	-2.8	16.8	1.984	0.202
	serum amylase	4 week	44	4	9.1	44	1	2.3	6.8	-2.9	16.5	1.908	0.360
		8 week	43	2	4.7	44	0	0.0	4.7	-1.6	10.9	2.095	0.241
		12 week	43	2	4.7	44	1	2.3	2.4	-5.3	10.0	0.370	0.616
	total cholesterol	4 week	44	3	6.8	44	4	9.1	-2.3	-13.6	9.0	0.155	1.000
		8 week	43	5	11.6	44	5	11.4	0.3	-13.1	13.7	0.001	1.000
		12 week	43	5	11.6	44	6	13.6	-2.0	-16.0	12.0	0.079	1.000
	HDL-cholesterol	4 week	44	0	0.0	44	1	2.3	-2.3	-6.7	2.2	1.011	1.000
		8 week	43	0	0.0	44	4	9.1	-9.1	-17.9	-0.3	4.097	0.116
		12 week	43	4	9.3	44	4	9.1	0.2	-11.9	12.4	0.001	1.000
	LDL-cholesterol	4 week	44	3	6.8	44	3	6.8	0.0	-10.5	10.5	0.000	1.000
		8 week	43	4	9.3	44	7	15.9	-6.6	-20.6	7.4	0.859	0.521
		12 week	43	5	11.6	44	4	9.1	2.5	-10.3	15.3	0.151	0.739
	triglyceride	4 week	44	3	6.8	44	4	9.1	-2.3	-13.6	9.0	0.155	1.000
		8 week	43	3	7.0	44	4	9.1	-2.1	-13.5	9.3	0.131	1.000
		12 week	43	2	4.7	44	2	4.5	0.1	-8.7	8.9	0.001	1.000
	glucose	4 week	44	1	2.3	44	1	2.3	0.0	-6.2	6.2	0.000	1.000
		8 week	43	1	2.3	44	2	4.5	-2.2	-9.9	5.4	0.322	1.000
		12 week	43	1	2.3	44	1	2.3	0.1	-6.2	6.4	0.000	1.000
	HbA1c (NGSP)	4 week	44	0	0.0	44	0	0.0	0.0	N.A.	N.A.	N.A.	N.A.
		8 week	43	0	0.0	44	0	0.0	0.0	N.A.	N.A.	N.A.	N.A.
		12 week	43	0	0.0	44	0	0.0	0.0	N.A.	N.A.	N.A.	N.A.
	gycoalbumin	4 week	44	1	2.3	44	3	6.8	-4.5	-13.2	4.2	1.048	0.616
		8 week	43	1	2.3	44	0	0.0	2.3	-2.2	6.8	1.035	0.494
		12 week	43	1	2.3	44	0	0.0	2.3	-2.2	6.8	1.035	0.494

n: number ;⊿: between-group difference (test food group - placebo group);95% CI -: 95% lower confidence interval/95% CI +: 95% upper confidence interval/chi 2: chi-square value; P: significant probability; 4 week: at test after 4 weeks of intake; 8 week: at test after 8 weeks of intake; 12 week : at test after 12 weeks of intake; N.A.: Not Available; *: comparison between groups using chi-square test
